# The Use of Platelet-Rich Fibrin-Coated Three-Dimensionally (3D) Printed Scaffolds in Salvage of Complex Hindfoot Cases

**DOI:** 10.3390/biomimetics10050269

**Published:** 2025-04-27

**Authors:** Ken Meng Tai, Justin Mooteeram, Anand Pillai

**Affiliations:** Wythenshawe Hospital, Manchester University NHS Foundation Trust (MFT), Southmoor Rd, Wythenshawe, Manchester M23 9LT, UK; justin.mooteeram@mft.nhs.uk

**Keywords:** 3D-printed implant, 3D-printed scaffold, hindfoot fusion, talar defect, RIA, AVN talus, PRF, platelet-rich fibrin, bioactive matrix

## Abstract

**Background:** Complex hindfoot pathologies involving critical-sized bone defects of the talus are difficult to manage. The current management involves arthrodesis and bone grafting with the defective talus, which have limitations in restoring structural integrity and functional goals. The advancement of 3D-printed scaffolds has opened new avenues to address such complex hindfoot pathologies, which may potentially improve treatment outcomes. The addition of platelet-rich fibrin further enhances healing potential. **Method:** This is a retrospective study involving six patients with severe talar bone loss secondary to osteomyelitis or avascular necrosis, where 3D-printed scaffolds coated with PRF were implemented in salvage surgery performed from 2023 to 2024. We intended to investigate the clinical outcomes in terms of healing time and union rate. Additionally, we evaluated the degree of deformity corrections and the patients’ clinical outcomes. **Results:** This study reports six complex reconstructions which achieved CT-confirmed union after a mean duration of 20.2 weeks. All patients were able to ambulate with full weight bearing after an average duration of 23.3 weeks. The patients demonstrated improved radiological parameters, VAS scores from 7.5 ± 1.4 points to 2.3 ± 1.2, and functional scores in all domains for AOFAS, FFI and SF-36. **Conclusion:** This study demonstrates the benefits of PRF-coated 3D-printed scaffolds in managing complex hindfoot cases, especially in the presence of significant bony defects. This modality has the potential to achieve a good union rate, near-anatomical correction and good functional outcomes.

## 1. Introduction

The hindfoot, which consists of the talus, calcaneus and the surrounding soft tissue, encompasses three important joints, namely, the subtalar, talonavicular and calcaneocuboid joints. This complex structure plays a crucial role in shock absorption, weight bearing and the gait cycle and affects the function of surrounding joints [1]. The rate of hindfoot pathologies is increasing along with the improving life expectancy, with a reported 13.4% of adults over the age of 50 years old experiencing hindfoot pain [2]. Complex hindfoot pathologies, which arise due to serious trauma, infections such as osteomyelitis and septic arthritis, avascular necrosis, degenerative arthritis, and failed reconstructive surgeries, entail formidable challenges for the orthopedic fraternity.

Talar deformity with associated bone loss is particularly arduous to manage. Talus integrity is crucial in maintaining hindfoot alignment and midfoot position through the transverse tarsal joint, which is a complex geometrical and biomechanical structure required for proper locomotion. Due to its innate lack of periosteal coverage, delicate blood supply, the absence of muscular attachment and the majority of its surface being enveloped by hyaline cartilage due to multiple joint articulations, it is susceptible to avascular necrosis (AVN) or osteonecrosis [3]. The prognosis of talar fracture is relatively poor, despite surgical management, with a reported overall incidence of AVN in 26.5 to 65% of cases, ankle arthritis in 51.7 to 98.0% of cases and subtalar arthritis in 45.0% of cases [4,5]. The long-term outcome of talus fracture after surgical treatment was reported to be moderate in terms of pain and disability score, with up to 41% of patients not being able to return to their daily activities [6].

Infection is another common cause of complex hindfoot pathologies. Septic arthritis causes significant articular cartilage damage due to the loss of chondrocytes. It was demonstrated that the 10-year survival of a native knee joint after an episode of septic arthritis was 65.5%, suggesting a secondary degenerative process [7]. The presence of osteomyelitis significantly worsens the prognosis, with direct bone damage caused by pathogens or radical debridement, including bone resection, in the treatment of this condition leading to bone loss and altered mechanical integrity. Such damage is also associated with multiple large cystic lesions and the possibility of collapse, which further limits surgical options [8]. Osteomyelitis of the hindfoot is reported to be associated with transtibial amputation in up to 52.2% of cases [9].

The common practice in managing complex hindfoot pathologies involves an arthrodesis, which may involve osteotomies, the removal of articular cartilage, bone grafting, and fixation with plates, screws, nails or external fixator devices. Such methods often fall short due to their failure to address patient-specific deformities. This may lead to complications, such as failure of correction, recurrence, implant failure and limb length discrepancy, in addition to arthrodesis-associated complications, which include nonunion, malunion, infection, hardware breakage or loosening, and amputation [10]. A recent systemic review on computed tomography scan confirmation of union reported an overall union rate of ankle arthrodesis of only 78.7% [11]. The occurrence of deformity is common after hindfoot fusion, with valgus tibiotalar tilt reported in up to 27% of cases [12]. Such complications are detrimental and may lead to impingement, adjacent joint arthritis and implant failure.

The emergence of 3D printing, also known as additive manufacturing, has opened up avenues in dealing with such complex hindfoot cases, especially in the presence of bone voids. This layer-by-layer process allows the manufacturing of scaffolds that specifically match patients’ unique deformities, allowing optimal fitting and mechanical stability. The use of titanium alloys, which are biocompatible and provide sufficient compressive strength and corrosive resistance, is suitable for load-bearing devices, reportedly achieving a similar elastic modulus to native bone [13]. Additionally, 3D printing allows the complex customization of implant designs while integrating porous structures and patterns which promote bone in-growth and integration [14]. It has been well established that porous implants with a size of 1000 μm achieve significantly better fusion rates in terms of vascularization and osteointegration compared to 500 μm and nonporous designs [15]. The roughened outer surfaces that are coupled to the bone promote osteoblast adhesion, which promotes osteointegration.

Three-dimensionally printed cages are particularly useful in managing critical-sized bone defects, typically defined as defects of more than 2.5 cm or 50% of the circumference of the involved bone, as union will not be achieved even with surgical stabilization [16]. The process of constructing such custom-made implants involves surgeons’ input and the expertise of bioengineers to match the defect size anatomically. This allows enhanced precision during surgery and reduces surgical time, which translates to better cost-effectiveness. Despite still being in its early stages, studies have reported good union rates with the use of 3D-printed cages for severe bone loss in foot and ankle cases, with 87% of patients reporting substantial pain and functional improvement [17].

There are, however, potential barriers to these new devices, with a study reporting that 33.3% of cases required a second surgery and that removal of implants due to nonunion was necessary in 25.6% of cases [18]. A systematic review involving spinal surgeries demonstrated subsidence of more than 3 mm in 11 out of 35 patients [19]. Given the scarcity of long-term studies of 3D-printed implants, there are concerns regarding osteointegration, wound healing, infection, rejection and allergic response. Platelet-rich fibrin (PRF), which is derived from the patient’s own blood, is autologous, biocompatible and rich in bioactive factors which could improve osteointegration and the union rate [20]. Autologous PRF produced by the Vivostat device was reported to have additional advantages in terms of the integration of tissues and the ability to coat implants while maintaining matrix integrity for up to 4 weeks [21]. This allows the use of antibiotics and additional bone grafts to be packed safely within the 3D-printed cage.

In this study, we intend to share our experience of implant design and the surgical process of this relatively new procedure. In addition, we intend to evaluate clinical outcomes in terms of wound healing rate, union rate, ambulatory status and complications encountered, as well as radiological outcomes in terms of sagittal and coronal parameters and functional outcomes with regard to changes in pain, functional and quality-of-life scores, in patients who underwent salvage surgery with PRF-coated 3D-printed scaffolds in complex hindfoot cases. We hypothesized that this new modality would ease surgical procedures and achieve good clinical outcomes, adequate radiological correction of deformity and acceptable functional outcomes.

## 2. Materials and Methods

This is a retrospective study involving 6 patients with complex hindfoot deformities surgically treated with PRF-coated 3D-printed scaffolds in Wythenshawe Hospital, Manchester University Foundation Trust, from 2023 to 2024. There were no exclusion criteria. All procedures were performed by a single board-certified foot and ankle consultant. The implants were manufactured by Meshworks^®^, UK, and conformed to the Medical Devices Directive 93/42 EEC (UK MDR 2002) and Medical Device Regulations (MDR 2017/745). The PRF matrixes utilized were ArthroZheal^®^ matrixes, prepared and applied through the Vivostat^®^ System (TRB Chemedica, Newcastle-under-Lyme, UK).

### 2.1. Preoperative Planning

CT scans were performed for surgical planning to determine the size and degree of the defect and the severity of deformity to assist in the implant design. An online teleconference between the surgical team, the Meshworks design engineer and the implant distributor was performed to discuss the required resection, the correction aim and implant design, and the configuration and position with 3D visualization. Auxiliary plastic guides that assist in bone resection and specific characterization of the cage, which includes the screw amount and direction, were mapped at this point. Implant design finalization was performed before manufacturing.

**Figure 1:**
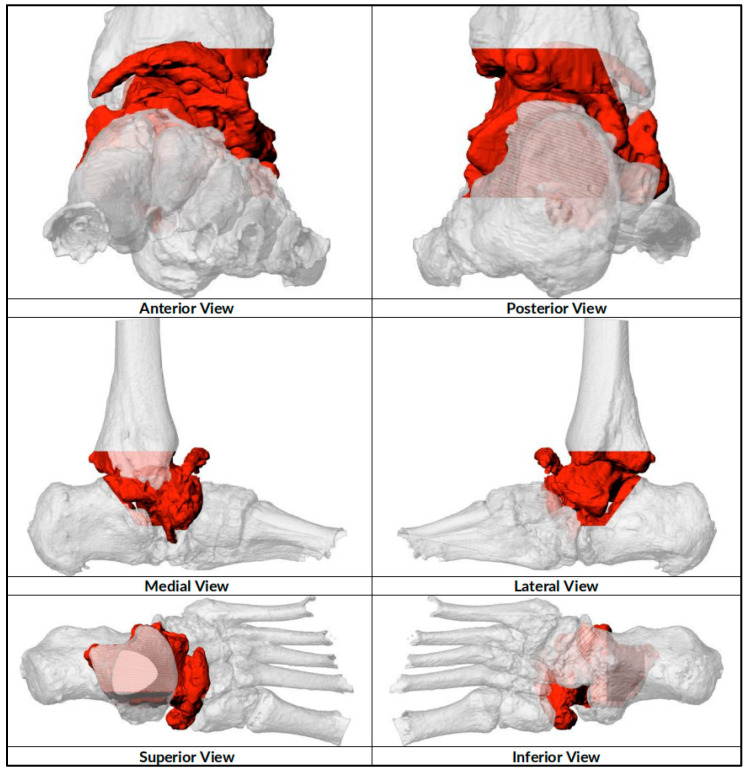
Preoperative resection planned for implant design. Red colored parts denote the planned resection area of the bones.

**Figure 2:**
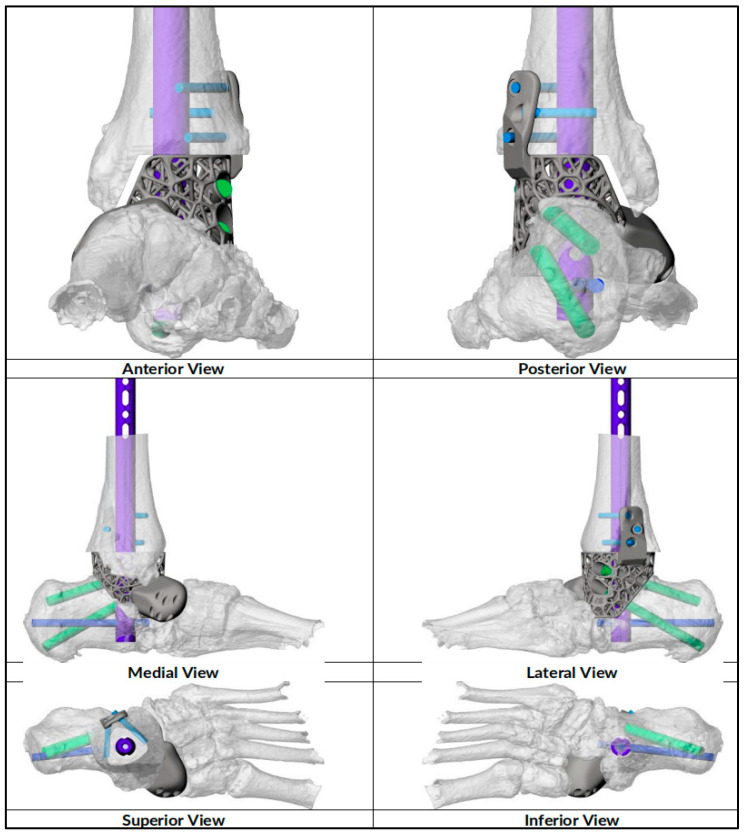
Preoperative reconstruction with implant position. Purple color denotes the hindfoot nail, blue color the nail screw and green color the cage incorporating screws.

**Figure 3:**
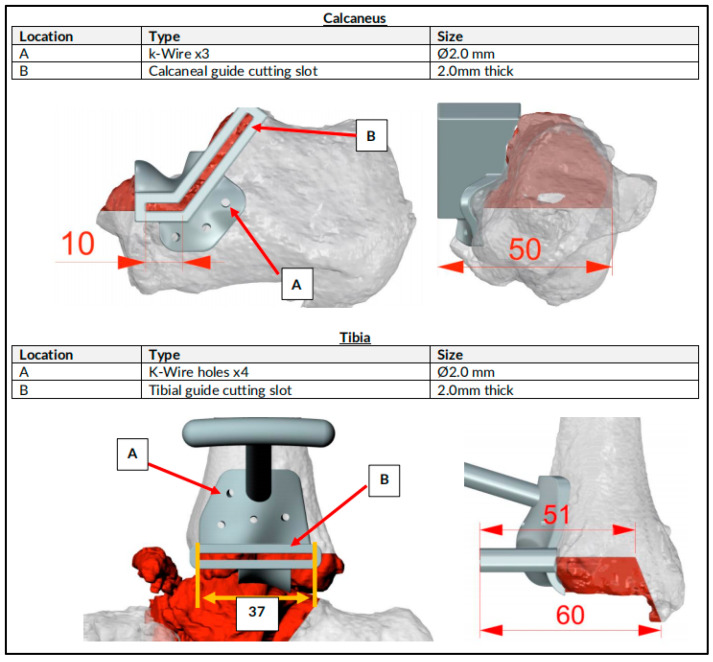
Preoperative plan of plastic guides for resection of distal tibia and calcaneum. Red colored parts denote the area of planned bone resection.

**Figure 4:**
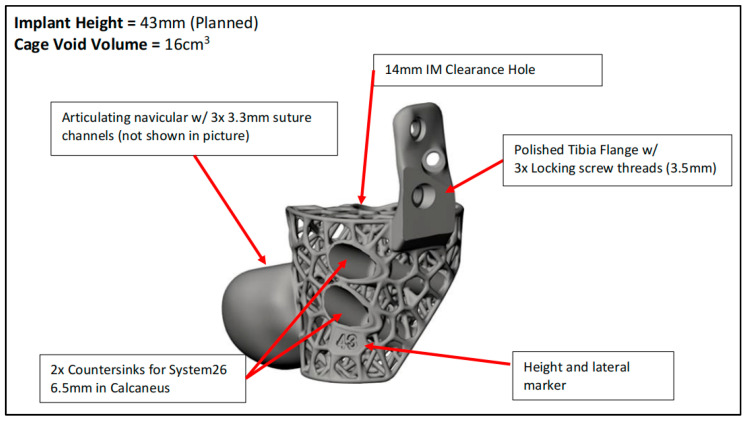
Finalized implant design.

The implants we utilized are produced at an ISO13485-accredited manufacturing facility in Oxford [22]. The implants were built via metal additive manufacturing, layer by layer, from titanium alloy powder by Selective Laser Melting (SLM) through a powder-bed fusion (PBF) process. The final implants used, including the cage, nail and screws, were made of the titanium alloy Ti6Al4V, which is also known as Grade 5 Titanium, containing 6% aluminum, 4% vanadium and titanium as the remainder, while the trials and guides were made of polyamide 12 (Nylon 12) powder (PA2200). Manufacturing to delivery for sterilization took approximately 2 weeks (Figure 1, Figure 2, Figure 3 and Figure 4).

### 2.2. Surgical Technique

**Figure 5:**
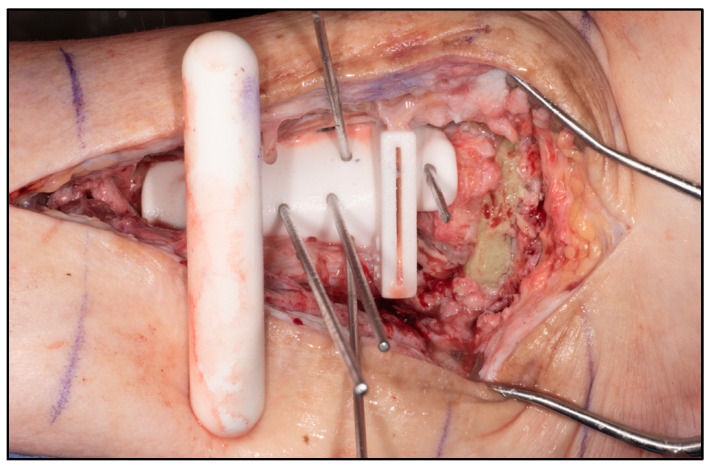
Bony cuts performed through plastic guides.

**Figure 6:**
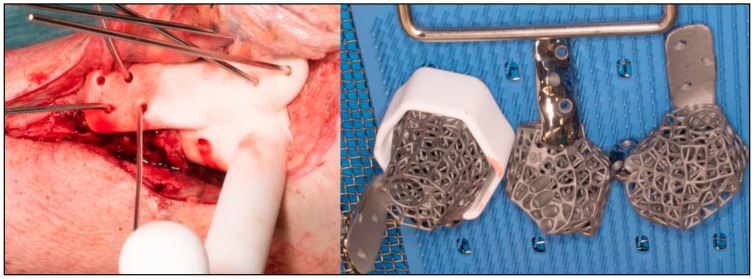
Trial performed with trial blocks with 3 different sizes of cages available.

The procedures were performed under general anesthesia with peripheral blocks. Prophylactic antibiotics (Teicoplanin and Gentamicin) were delivered during induction. The patients were first positioned supinely on a radiolucent traction table for the reamer–irrigator–aspirator (RIA) procedure to obtain sufficient autologous bone grafts. They were then repositioned supinely on a radiolucent table with their foot at the lower end of the table. A high thigh tourniquet was applied and inflated after standard prepping and draping. Surgical approaches were dependent on previous scarring, major pathological side effects and additional deformities which may have required supplementary incisions. Talectomy was performed, and bony resection of the distal tibia and calcaneum was carried out based on preoperative planning. Bony resection was completed with the help of a specific auxiliary guide matching the implant geometry (Figure 5). Trials were performed with 3 sizes of 3D-printed trial blocks, and the ones that best fitted positionally were chosen (Figure 6). Wound washout was performed, followed by the insertion of multiple drill holes at fusion sites with K-wires for improved osteointegration.

**Figure 7:**
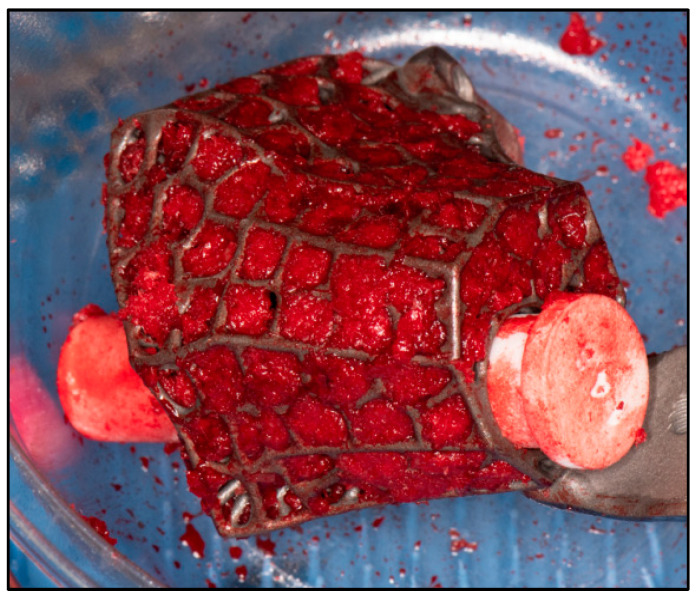
Cage filled with autologous bone graft harvested from RIA.

**Figure 8:**
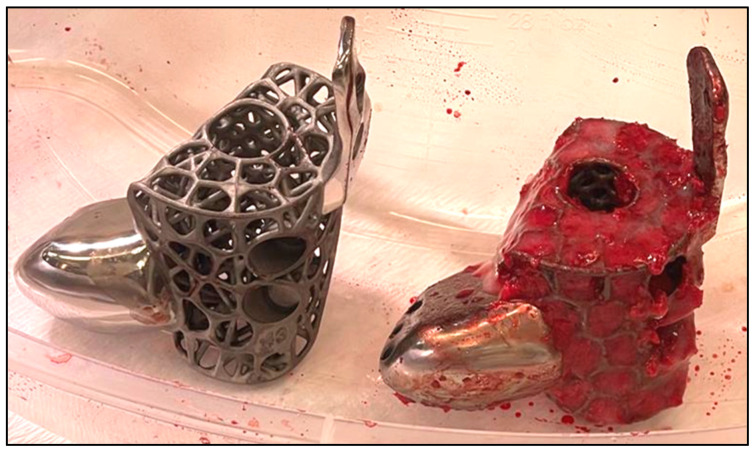
Pre- and post filling with bone graft and application of Arthrozheal matrix.

For implants that incorporate the hindfoot nail, the guidewire was passed through the calcaneus into a cylindrical hole on the 3D trial block to the tibia before reaming in ascending sizes was performed. The chosen 3D-printed keystone talus cage was filled with an autologous bone graft harvested from RIA. (Figure 7). Arthrozheal matrixes were then applied to the surface of the keystone talus with the Vivostat^®^ Applicator Unit (Figure 8). The nail guidewire was removed, and the keystone talus was then inserted into the prepared space. The guidewire was reinserted, followed by the insertion of the desired tibiotalocalcaneal nail (Oxbridge ankle fusion nail) while maintaining the optimum hindfoot position. Proximal locking screws were inserted through the holes of the hindfoot nail. Further compression was performed through attachment of the jig through the nail to improve the bony interface before the insertion of distal locking screws through the calcaneum. Additional screws were inserted from the cage into the calcaneum when necessary. Locking screws were supplemented for implants which incorporated tibial or navicular flanges. Wounds were closed in layers, followed by sterile dressing, padding and application of a below-knee cast.

### 2.3. Postoperative Care

The patients were admitted with leg elevation until wound inspection on the third postoperative day, when new full casts were applied. Teicoplanin was administered for 3 days before discharge with strict non-weight-bearing and leg-elevation instructions. Deep vein thrombosis prophylaxis in the form of Dalteparin was supplied until the patients were able to partially weight bear, typically more than 3 months after surgery. Wound inspection was performed every 2 weeks until full healing, and casts were kept for a minimum of 3 months. Plain radiographs and CT scans were performed at 3 months and 6 months to confirm union before the patients were allowed to fully weight bear. The patients were followed up for a minimum of 1 year.

### 2.4. Data Collection

Information on patient demographics, comorbidities, clinical features, investigations, surgical procedures and outcomes was collected from the hospital electronic database system. During follow-ups, the patients were monitored with Visual Analogue Scale (VAS) and American Orthopaedic Foot and Ankle Society (AOFAS) scores. They were then given the 36-Item Short Form Survey Instrument (SF-36) and Foot Function Index (FFI) questionnaires to monitor functional outcomes. Radiographic parameters from plain radiographs were documented before and after surgery to identify the degree of correction.

## 3. Results

Our study involved six patients with a mean age of 64.3 years (range: 59 to 78 years), five (83.3%) males and one (16.7%) female. The body mass index (BMI) average was 29.5 (26.5 to 35.4), with four overweight and two obese. Three patients were smokers, and three were nonsmokers. Two patients had peripheral arterial disease (PAD), one with involvement at the level of the tibioperoneal trunk and the other at the level of the posterior tibial artery. One patient suffered from Charcot neuroarthropathy, while three (50%) patients had chronic kidney disease. Glycated hemoglobin (HbA1c) levels averaged at 45.6 mmol/mol (range: 36–70). The preoperative hemoglobin level mean was 120.5 g/L (range: 105–146), while the C-reactive protein average was 9.3 (range: 6 to 16).

Three (50%) patients suffered from osteomyelitis secondary to infected implants, while the other three (50%) cases involved post-traumatic arthritis secondary to avascular necrosis (AVN) of the talus. The three cases with osteomyelitis underwent two-stage surgery, where the first stage involved removal of the implants, methodical debridement and antibiotic cement spacer application for at least 3 months before the 3D-printed scaffold fixation. The other three patients underwent single-stage surgery after confirming the absence of infection. With regard to prior surgeries, one patient had five, two cases had three, one had single surgery and two patients had none. The surgical approaches were variable, with two cases involving medial incision alone, two involving a lateral approach alone and two involving lateral and dorsomedial approaches. All cases involved a 3D-printed keystone talus, with five including a hindfoot nail, one involving a total articulating navicular extension with a tibial flange, two involving tibial and navicular flanges and two involving a navicular flange. The keystone cage incorporated custom-planned clearance holes for additional screws, with two additional subtalar screws implemented in five cases, two screws to the talus in one case, one screw to the tibia in one case and one screw to the navicular flange in one case. All cases involved packing of autologous bone grafts harvested from RIA, followed by PRF application.

**Figure 9:**
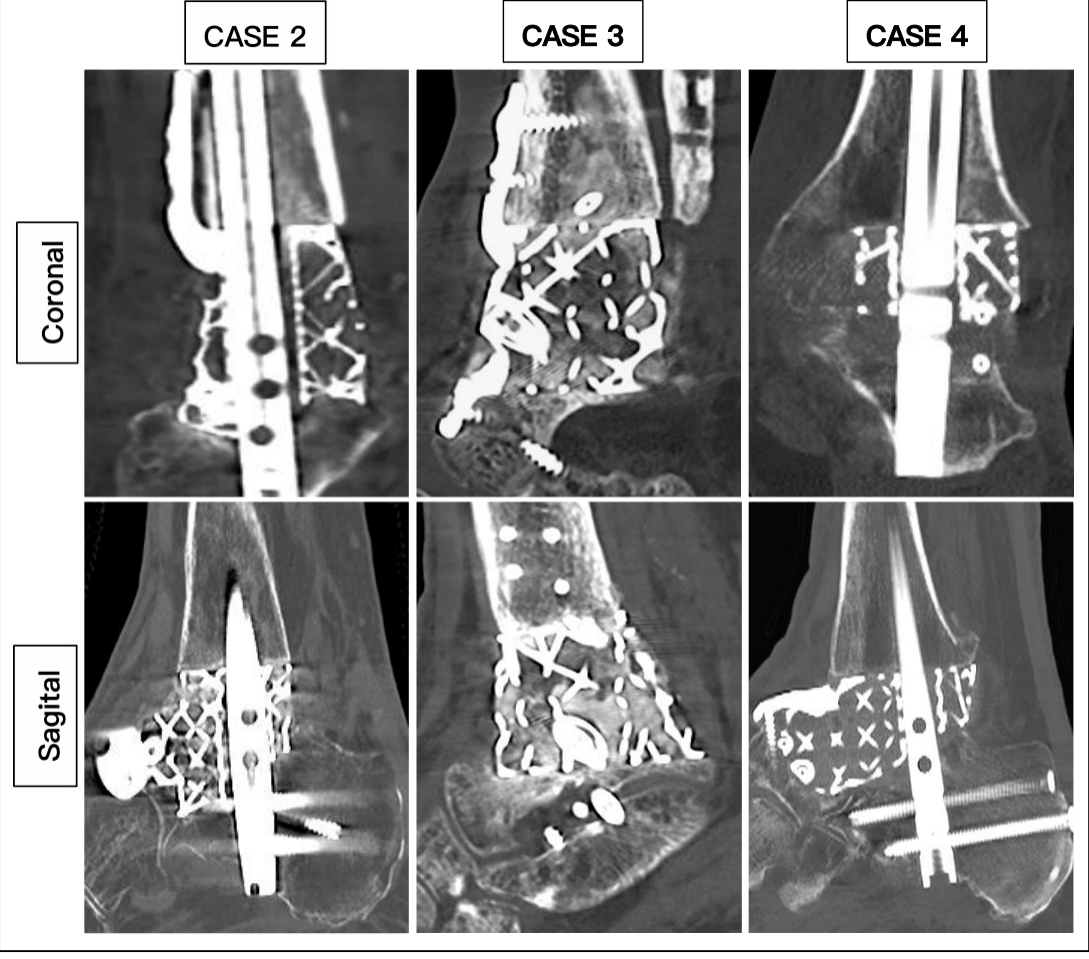
Demonstration of union from CT scans.

The average duration of surgery was 207 min (range: 162 to 255). None of the patients required a blood transfusion. All the patients were admitted for 3 days. The mean duration of complete wound healing was 34.7 days (range: 20 to 45 days). All patients (100%) achieved union based on CT scans, with a mean duration of 20.2 weeks (range: 14 to 27 weeks) (Figure 9). All patients were able to ambulate in full weight bearing after an average duration of 23.3 weeks (range: 16 to 29 weeks). One patient sustained a superficial wound infection and was treated with oral antibiotics, which resolved the infection after 2 weeks (Table 1).

**Figure 10:**
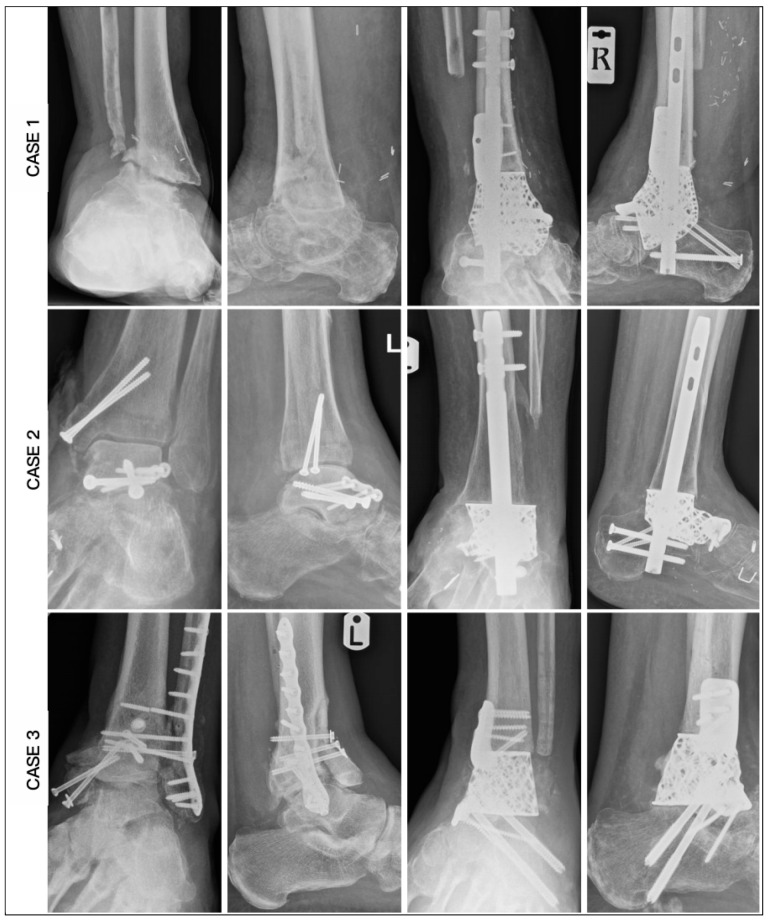
Plain radiographs of AP and lateral pre- and post-fixation for cases 1 to 3.

**Figure 11:**
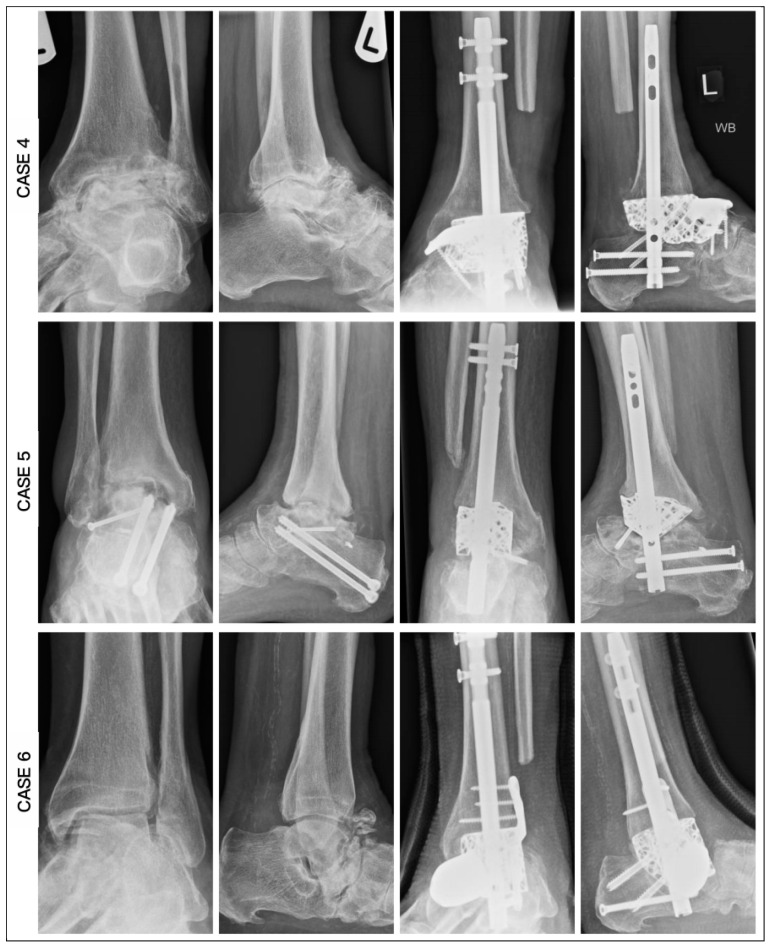
Plain radiographs of AP and lateral pre- and post-fixation for cases 4 to 6.

Radiographic measurements were taken pre- and postoperatively (Figure 10 and Figure 11). From the AP radiographs, the preoperative tibiocalcaneal angle, tibiocalcaneal distance, foot height and tibiotalar angle averaged at 14.5 degrees (range: 6.8 degrees varus to 38.4 degrees), 25.7 mm (range: 6.2 to 53.2), 50.3 mm (range: 38.3 to 58.1) and 6.8 degrees (range: 6.1 degrees varus to 16.1 degrees), and the postoperative means were 4.5 degrees (range: 3.0 to 6.2), 8.0 mm (range: 4.8 to 11.7), 56.8 mm (range: 50.1 to 74.3) and 0.8 degrees (range: 0.2 to 1.4), with difference means of 14.0 degrees (range: 6.3 to 32.0), 20.5 mm (range: 9.8 to 44.1), 7.3 mm (range: 0.2 to 19.5) and 7.6 degrees (range: 0.5 to 12.4). The lateral radiographs revealed an average preoperative lateral distal tibia angle, lateral tibiotalar angle, Meary angle, calcaneal inclination angle, navicular height and plantigrade angle of 88.7 degrees (range: 76.6 to 101.5), 84.5 degrees (range: 59.1 to 107.6), 9.9 degrees (range: 2.3 to 24.1), 19.0 degrees (range: 4.7 to 37.1), 34.3 mm (range: 17.4 to 44.8) and 88.8 degrees (range: 85.1 to 93.7), while the postoperative means were 88.6 degrees (range: 85.3 to 92.7), 70.4 degrees (range: 67.3 to 72.1), 3.0 degrees (range: 1.8 to 5.3), 20.0 degrees (range: 14.8 to 22.6), 40.6 mm (range: 35.9 to 46.5) and 89.4 degrees (range: 86.9 to 92.6), with mean differences of 7.6 degrees (range: 0.5 to 12.4), 19.4 degrees (range: 4.0 to 40.3), 6.9 degrees (range: 0.3 to 18.8), 8.2 degrees (range: 0.3 to 16.0), 7.5 mm (range: 1.7 to 20.7) and 2.6 degrees (range: 0.3 to 5.2) (Table 2).

The preoperative VAS and AOFAS scores averaged at 7.5 (range: 5 to 9) and 13.5 (range: 7 to 27), while the postoperative means were 2.33 (range: 1 to 4) and 69 (range: 62 to 86), with average improvements of 5.2 (range: 4 to 6) and 55.5 (range: 51 to 59). The FFI preoperative average overall, pain, disability and activity limitation scales were 91.5% (range: 83 to 96), 78.7% (range: 46 to 100), 96% (range: 90 to 100) and 99.5% (range: 97 to 100) compared to postoperative means of 39.3% (range: 19 to 51), 24% (range: 6 to 32), 50.2% (range: 30 to 63) and 31.5% (range: 10 to 53), with average improvements of 52.2% (range: 45 to 69), 55% (range: 26 to 68), 45.8% (range: 32 to 70) and 68% (range: 47 to 90). The reported SF-36 preoperative averages for each domain involving physical functioning; role limitations due to physical health; role limitations due to emotional problems, energy and fatigue; emotional well-being; social functioning; pain; and general health were 10% (range: 5 to 15), 0%, 11.1% (range: 0 to 33.3), 19.2% (range: 5 to 35), 23.3 (range: 16 to 36), 12.5% (range: 0 to 25), 18.3 (range: 0 to 45), 20.8% (range: 5 to 35) and 20.8 (range: 0 to 25), while the postoperative means were 56.7% (range: 45 to 85), 54.2% (range: 25 to 75), 77.8% (range: 66.7 to 100), 65% (range: 45 to 80), 75.3% (range: 60 to 84), 66.7% (range: 50 to 87.5), 79.2 (range: 67.5 to 87.5), 69.3% (range: 50 to 85) and 87.5% (range: 75 to 100), with average improvements of 46.7% (range: 40 to 75), 54.2 (range: 25 to 75), 66.7% (range: 33.4 to 100), 45.8% (range: 35 to 60), 52% (range: 40 to 68), 54.2% (range: 25 to 75), 60.8 (range: 42.5 to 77.5), 48.5% (range: 41 to 55) and 66.7% (range: 50 to 100) (Table 3).

## 4. Discussion

Hindfoot deformities with the presence of a nonsalvageable talus are complex to manage, as the talus functions as a keystone structure of the ankle. The treatment plan often relies on the architecture of the remaining talus. As patients often present with secondary arthritis to ankle and subtalar arthritis, treatment options become severely limited. The emergence of 3D-printed scaffolds has opened new options with custom-fit cages which accommodate arthrodesis modalities. These custom-made implants have been reported to achieve far superior union rates of around 75% compared to femoral head allografts [23].

Union is the most important outcome, as it often defines the success of any fusion surgery. The majority of implant-related studies focus on fusion rates and durations due to significant functional loss associated with nonunion. The definition of union, however, remains controversial, as the traditional use of plain radiographs to determine union is not accurate because it does not correlate with the patient’s functional outcome. A study comparing the use of radiographs and CT scans reported significant differences between the two, with radiographs ambiguously reporting much higher union rates [24]. The definition of union based on CT scans is, however, variable, ranging from 25 to 70% of osseous bridging, with the majority of studies delineating it as 50% [25,26]. Additionally, studies have reported better clinical outcomes with CT-defined osseous bridging of more than 25–50% compared to rates below 25% [27]. In our study, we defined union as osseous bridging of more than 50% from CT scans with a good functional outcome, primarily, the ambulatory status.

The reported union rates with the use of 3D-printed scaffolds for ankle arthrodesis range from 84 to 100% [28,29]. Our study demonstrates a similar union rate of 100%. The duration to union has been reported in a systemic review to range from 4 to 6 months, with a mean of 5.3 months [30]. Our study demonstrates a similar duration to union, with an average of 20.2 weeks, which translates to 4.6 months. Compared to the majority of studies, in which autografts and allografts are used, one study reported a union rate of 100%, with functional union after 2.6 months, with the application of RIA [31]. We postulate that the use of a PRF matrix with RIA improves the union rate and the duration to union.

Despite 3D-printed scaffolds being a relatively new modality, the reported functional outcomes have been promising. Functional outcomes are crucial to determine the effectiveness of such new implants, as we can objectively compare the pre- and postoperative differences. With regard to pain scores, most studies report significant improvements, with one study demonstrating a reduction in VAS from 6.6 ± 2.9 points to 2.0 ± 1.7 points. Our study reported a similar outcome, with an improvement in VAS from 7.5 ± 1.4 to 2.3 ± 1.2 points. Several studies have proclaimed improvements in functional outcomes with the use of AOFAS, FFI and SF-36 scoring in all domains, similar to our study [32,33]. Overall improvement in most available functional scores could raise the confidence of surgeons to pursue this new technique as a limb-salvage option.

Radiographic parameters are crucial in the field of orthopedics, as they allow the quantification of the severity of a deformity and proper surgical planning. As the aim of surgery is to achieve near-anatomical reduction, these parameters are valuable in determining postoperative improvements. There are, however, limited studies reporting degrees of correction, with one study demonstrating a mean coronal correction of 25 degrees and sagittal correction of 6 degrees. [29]. In the present study, we demonstrated similar improvement but included all necessary parameters.

We acknowledge that there are several limitations to our study. The sample size in this study was only six patients, as we were investigating a new modality to treat this rare complication. This was a retrospective study describing the use of a single technique, namely, the use of PRF-coated 3D-printed scaffolds in managing complex hindfoot deformities with significant bony defects. A comparative study reflecting the clinical outcomes would be significant. Although we reported all the cases where this surgery was performed, the gender distribution of five males and one female could be a potential source of bias. A longer follow-up duration would improve the assessment of long-term outcomes.

## 5. Conclusions

This study intended to demonstrate the potential of PRF-coated 3D-printed scaffolds in managing complex hindfoot cases, especially in the presence of significant bone defects. This modality demonstrated a good union rate, near-anatomical correction and good functional outcomes, without any significant complications. The addition of PRF can potentially enhance the union rate and time to complete osteointegration. Further comparative studies with larger numbers of patients are required to determine statistical significance to improve the quality of care for such cases.

## Figures and Tables

**Table 1:** Demographics, surgical details and clinical outcomes.

CASE	1	2	3	4	5	6
Age	62	59	60	60	78	67
Diagnosis	OM	OM	OM	AVN	AVN	AVN
Smoking	No	Yes	Yes	No	Yes	No
BMI	26.5	27.4	27.4	28.2	32.1	35.4
Medical	Nil	IHD, CKD, PAD	CKD	CKD, PAD	DM	IHD, DM, CKD, CN
HbA1c	36	39	39	41	49	70
CRP	8	7	16	9	10	6
Hb (g/L)	113	105	132	146	113	114
Past surgery	5	3	3	0	1	0
Modification	Tibial and Navicular Flange, Nail	Navicular Flange, Nail	Tibial and Navicular Flange	Navicular Flange, Nail	Nail	Articulating Navicular Extension, Tibial Flange, Nail
Duration of surgery (min)	255	220	162	210	190	205
Wound Healing (days)	42	40	20	29	32	45
Union (weeks)	25	19	16	14	27	20
Ambulation (weeks)	28	21	18	16	29	22

Abbreviations: OM, Osteomyelitis of Talus; AVN, Avascular Necrosis; BMI, Body Mass Index; IHD, Ischemic Heart Disease; CKD, Chronic Kidney Disease; PAD, Peripheral Arterial Disease; DM, Diabetes Mellitus; CN, Charcot Neuroarthropathy.

**Table 2:** Radiographic parameters pre- and post-surgery.

CASE	1	2	3	4	5	6
Tibiocalcaneal angle (pre)	38.4	18.6	12.4	−6.8	13.8	10.5
Tibiocalcaneal angle (post)	6.2	3.0	3.4	4.2	6.1	4.2
Difference	32.2	15.6	9.0	11	7.7	6.3
Tibiotalar angle (pre)	16.1	4.4	12.4	−6.1	11.9	2.1
Tibiotalar angle (post)	1.4	1.2	0.9	0.2	0.5	0.7
Difference	14.7	3.2	11.5	6.3	11.4	2.0
Tibiocalcaneal distance (pre)	53.2	24.0	24.0	6.2	27.7	18.9
Tibiocalcaneal distance (post)	9.1	7.2	4.8	6.0	11.7	9.1
Difference	44.1	16.8	19.2	17.2	16.0	9.8
Foot height (pre)	43.8	58.1	54.8	54.1	52.8	38.3
Foot height (post)	51.9	58.3	74.3	51.5	54.6	50.1
Difference	8.1	0.2	19.5	2.6	1.8	11.8
Lateral distal tibial angle (pre)	81.2	87.5	76.6	97.7	101.5	87.8
Lateral distal tibial angle (post)	88.9	87	86.6	85.3	91.3	92.7
Difference	7.7	0.5	10.0	12.4	10.2	4.9
Lateral tibiotalar angle (pre)	87.8	67.1	107.6	81.5	103.6	59.1
Lateral tibiotalar angle (post)	69.9	71.1	67.3	72.1	70.8	71.2
Difference	17.9	4.0	40.3	9.4	32.8	12.1
Meary angle (pre)	8.0	10.1	2.3	3.2	24.1	11.4
Meary angle (post)	3.1	2.0	2.0	1.8	5.3	3.6
Difference	4.8	8.3	0.3	1.4	18.8	7.8
Calcaneal inclination (pre)	13.7	37.1	14.5	16.1	27.9	4.7
Calcaneal inclination (post)	22.5	21.1	14.8	22.6	22.1	16.7
Difference	8.8	16.0	0.3	6.2	5.8	12
Navicular height (pre)	33.1	39.3	44.8	37.5	33.4	17.4
Navicular height (post)	41.4	35.9	46.5	41.6	40.1	38.1
Difference	8.3	3.4	1.7	4.1	6.7	20.7
Plantigrade angle (pre)	85.1	93.7	86.9	90.0	89.8	87.4
Plantigrade angle (post)	86.9	88.7	89.4	89.5	89.5	92.6
Difference	1.8	5.0	2.5	0.5	0.3	5.2

**Table 3:** Clinical and functional outcomes pre- and post-surgery.

CASE	1	2	3	4	5	6	Mean Difference
**Pain Score**							
VAS (pre)	8	7	8	9	8	5	5.2 ± 0.8
VAS (post)	2	1	3	4	3	1	
**Functional Score**							
AOFAS (pre)	20	27	8	10	9	7	55.5 ± 3.2
AOFAS (post)	71	86	63	66	62	66	
FFI (pre)	93	88	96	95	94	83	52.2 ± 9.1
FFI (post)	39	19	51	48	41	38	
Pain (pre)	84	60	88	100	94	46	55.0 ± 14.8
Pain (post)	26	6	28	32	32	20	
Disability (pre)	96	100	100	90	92	98	45.8 ± 13.4
Disability (post)	49	30	63	58	52	49	
AL (pre)	100	100	100	100	97	100	68.0 ± 15.0
AL (post)	30	10	53	43	20	33	
**Quality of Life (SF-36)**							
PF (pre)	10	10	5	10	10	15	46.7 ± 14.0
PF (post)	55	85	45	50	50	55	
RP (pre)	0	0	0	0	0	0	54.2 ± 18.8
RP (post)	50	75	25	50	50	75	
RE (pre)	0	33.3	0	0	0	33.3	66.7 ± 21.1
RE (post)	66.7	100	66.7	66.7	100	66.7	
EF (pre)	5	35	10	20	10	35	45.8 ± 10.2
EF (post)	65	80	45	65	65	70	
EW (pre)	20	36	20	24	24	16	52.0 ± 10.1
EW (post)	80	84	60	72	72	84	
SF (pre)	0	25	0	25	12.5	12.5	54.0 ± 17.0
SF (post)	75	87.5	50	50	75	62.5	
Pain (pre)	22.5	32.5	0	0	10	45	60.8 ± 15.6
Pain (post)	67.5	87.5	77.5	77.5	77.5	87.5	
GH (pre)	30	35	5	15	15	25	49.0 ± 5.8
GH (post)	71	85	50	70	70	70	

Abbreviations: VAS, Visual Analogue Scale; AOFAS, American Orthopaedic Foot and Ankle Society; FFI, Foot Function Index; AL, Activity Limitation; SF-36, Short Form Health Survey; PF, Physical Functioning; RP, Role Limitations due to Physical Health; RE, Role Limitations due to Emotional Problems; EF, Energy/Fatigue; EW, Emotional Well-Being; SF, Social Functioning; GH, General Health.

## Data Availability

No data are provided due to privacy restrictions.
